# Application of ALSO course in standardized training Resident in Obstetric

**DOI:** 10.1186/s12909-024-05126-6

**Published:** 2024-02-16

**Authors:** Li Zhiyue, Lu Dan

**Affiliations:** Clinical Medical College of Yangzhou University, 225001 Yangzhou, China

**Keywords:** Obstetrics, Standardization training of residents, ALSO teaching method, Clinical skill and operation

## Abstract

**Objective:**

To explore the teaching effect of Advanced Life Support in Obstetrics (ALSO) Course in the standardized training resident in obstetric.

**Methods:**

60 residents of obstetrics from January 2021 to December 2022 were randomly divided into two groups, observation group and control group. The experimental group used ALSO teaching method, and the control group used traditional teaching method. The teaching effect was evaluated by theoretical examination, direct observation of procedural skills (DOPS) scale and mini clinical evaluation (Mini-CEX) scale.

**Results:**

The theoretical achievements of the observation group were significantly higher than that of the control group (*P* < 0.05). The pre-procedural preparation, safe analgesia, technique of procedure, aseptic technique, seeks help when necessary, post-procedural management, communication skills, humanistic care and overall performance score of the DOPS in the experimental group were higher than those in the control group (*P* < 0.05). The organization efficiency, humanistic qualities, manipulative skills, clinical judgment, medical interviewing skills and overall clinical competence score of the Mini-CEX in the experimental group were higher than those in the control group (*P* < 0.05).

**Conclusions:**

ALSO teaching method has an ideal effect in the standardization training of residents of obstetrics, indicating the prospect of active in-depth research and expanded application.

**Supplementary Information:**

The online version contains supplementary material available at 10.1186/s12909-024-05126-6.

## Introduction

Standardized training of residents, as an important part of postgraduate medical education, is the necessary stage of training highly competent professionals [1]. Obstetrics and gynecology, particularly obstetrics, is characterized by high risk, emergency conditions, and strong integration; therefore, residency plays an essential role in the development of a clinical medicine graduate into an obstetrics clinician [2, 3]. The traditional model of medical education is still founded on classroom lectures, and trainees are frequently passive recipients, which is not conducive to the development of their clinical problem analysis and practical skills [4]. The Advanced Life Support in Obstetrics (ALSO) course is a clinically based, interprofessional, multidisciplinary educational program that aims to promote obstetricians’ learning of knowledge and skills through training in the most recent international obstetrical knowledge and skills, using rational methods to effectively manage critical, severe, and emergency obstetrical conditions, and standardizing obstetrical operations, with the ultimate goal of enhancing obstetric care [5]. In this study, we analyzed the impact of a standardized obstetric advanced life support course on the standardized training of obstetric residents and provided a foundation for future in-depth research on the standardization of resident training.

## Materials and methods

### Participants

Sixty participants were all resident doctors who received Standardized Residency Training in Obstetrics and Gynecology in our department between January 2021 and December 2022 were used as study subjects and were divided into a control group (*n* = 30) and an observation group (*n* = 30) using the random number table method, with the control group receiving the traditional teaching method and the observation group receiving the ALSO teaching method; both groups had no prior work experience. In terms of gender, age, education, and training year, there was no statistically significant difference (*P* > 0.05) between the two categories.

### Teaching methods

The control group trainees were trained using traditional teaching methods, with the instructor demonstrating the theoretical knowledge of the selected teaching content via blackboard-writing, teaching aids, multimedia, etc., and then leading the residents to perform skill demonstrations in the clinical skill center, such as physical examination and history collection, etc. The residents practiced independently after the demonstration.

The observation group adopted the ALSO teaching method and the specific content of ALSO courses for the residents in the observation group is shown in Table S1. The courses were divided into a theoretical class and a hands-on practical component, with required reading and lectures, slide presentations, and practical exercises for each chapter.

With the example of shoulder dystocia, the ALSO teaching procedure was introduced. Firstly, the instructor introduces the participants to the learning objectives of the course, including recognition of risk factors, identification and standardized management for shoulder dystocia. Then, the instructor correctly demonstrates the administration of shoulder dystocia using the HELPERR mnemonic, consist of H (call for help), E (evaluate for episiotomy), L (legs), P (suprapubic pressure), E (enter maneuvers), R (remove the posterior arm), R (roll the patient). Thereafter, clinical scenarios were set up for students to practice on simulated individuals. Ultimately, the participants reviewed the process based on the contents of the records, and then the instructor conducts an evaluation, highlighting the deficiencies and strengths, answering any concerns raised by the participants, and providing a general assessment. All of the interviews were recorded.

### Evaluation of teaching effect

After the training, theoretical and technological assessment were conducted, including, (1) Theoretical assessment of obstetrics: The theory test is mainly composed of objective questions, which is centered on clinical practical application. The full score of the test was 100 points, including 80 points for eighty single-choice questions and 20 points for four short-answer questions. Spearman correlation coefficient was calculated to assess the split-half reliability for the theoretical test. The split-half coefficient for the theoretical test was 0.803, indicating the designed theoretical test possessed high reliability. (2) Operational assessment of obstetrics: Direct Observation of Procedural Skills (DOPS) rating scales were used for assessment of procedural skills (including understanding of indications, obtaining informed consent, preparation of pre-procedure, appropriate level of pain relief, technical ability, aseptic technique, seeking help where appropriate, post-procedure management, communication skills, consideration of patient and overall ability to perform the procedure). Mini Clinical Evaluation (Mini-CEX) were used for clinical comprehensive ability (including history taking, physical examination skills, organization efficiency, humanistic qualities, clinical operating ability, clinical judgement, health education, communication skills and overall clinical competency). Both two tests were marked on a 9-point scale (1–3: to be strengthened, 4–6: up to standard, 7–9: excellent).

### Ethics

The study protocol was approved by the Ethics Committee of Northern Jiangsu People’s Hospital (ethical review: 202,078). All subjects provided informed consent prior to their inclusion in the study. All the procedures performed in this study were in accordance with the principles of the Declaration of Helsinki.

### Statistical analysis

SPSS 22.0 (SPSS Inc, Chicago, IL) was used for the data Analysis. The counting data were expressed in frequencies, and the measurement data were expressed as mean ± standard deviation. Prior to the statistical analysis, the comparison data were normally distributed. Subsequently, count data were compared using Chi-square test and measurement data were compared using the independent sample t test for the two groups. *P* < 0.05 was considered a statistically significant difference.

## Results

### Basic characteristics and information

As shown in Table 1, there were no significant differences between control group and observation group in terms of gender, age, educational background or grades (*P* > 0.05).

**Table 1:** The basic characteristics of all the participants

Item		Traditional group	ALSO group	*P* value
Gender	Male Female	6 24	4 26	0.370
Age (in years)		22.5 ± 1.2	21.8 ± 0.7	0.423
educational background	Bachelor master	8 22	11 19	0.296
Grade	1st year 2nd year	15 15	18 12	0.183

Note: Independent sample t test (Age) and Fisher exact test (Gender, educational background, Grade) are used in Table 1

### Theoretical assessment of obstetrics

The theoretical knowledge scores in the ALSO group were significantly higher than those in the traditional group (*P* < 0.05, Table 2).

**Table 2:** The assessment score of the residents in both group (${\rm{\bar x}} \pm {\rm{s}}$)

Group	n	theoretical knowledge score
Traditional group	30	83.6 ± 3.2
ALSO group	30	92.4 ± 2.7
*P* value		0.002

Note: Chi-square test is used in Table 2

### Comparison of items in DOPS tests in the traditional group and ALSO group

Based on the assessment results, the residents in ALSO group performed significantly better than those in traditional group in terms of DOPS tests in pre-procedural preparation, safe analgesia, technique of procedure, aseptic technique, seeks help when necessary, post-procedural management, communication skills, humanistic care and overall performance (*P* < 0.05, Table 3).

**Table 3:** Comparison of DOPS score by traditional group and ALSO group (${\rm{\bar x}} \pm {\rm{s}}$)

Obstetrics DOPS	Traditional group	ALSO group	*P* value
understanding of indications	6.46 ± 1.72	6.85 ± 1.43	0.625
obtaining informed consent	6.12 ± 1.61	6.91 ± 1.75	0.087
preparation of pre-procedure	4.76 ± 1.07	6.79 ± 1.02	0.011
appropriate level of pain relief	4.28 ± 1.32	6.43 ± 1.53	0.008
technical ability	4.34 ± 1.25	5.64 ± 1.41	0.004
aseptic technique	3.57 ± 1.41	5.87 ± 1.32	0.039
seeking help where appropriate	4.61 ± 0.95	5.64 ± 1.23	0.000
post-procedure management	5.02 ± 0.86	5.92 ± 0.89	0.020
communication skills	5.28 ± 1.21	6.77 ± 1.15	0.014
consideration of patient	4.73 ± 1.18	5.81 ± 1.21	0.003
overall ability to perform the procedure	5.07 ± 1.12	6.47 ± 1.72	0.001

Note: Independent sample t test is used in Table 3

### Comparison of items in Mini-CEX tests in the traditional group and ALSO group

The results of the Mini-CEX assessment was shown in Table 4. The performance of ALSO group was better than traditional group in most aspects including organization efficiency, humanistic qualities, clinical operating ability, clinical judgement, health education, communication skills and overall clinical competency (*P* < 0.05).

**Table 4:** Comparison of Mini-CEX score by traditional group and ALSO group (${\rm{\bar x}} \pm {\rm{s}}$)

Obstetrics Mini-CEX	Traditional group	ALSO group	*P* value
history taking	7.17 ± 1.81	7.35 ± 1.43	0.082
physical examination skills	7.02 ± 1.56	7.32 ± 1.14	0.129
organization efficiency	4.04 ± 1.16	6.38 ± 1.72	0.012
humanistic qualities	4.63 ± 1.52	6.89 ± 1.47	0.003
clinical operating ability	6.79 ± 1.37	7.22 ± 1.21	0.020
clinical judgement	6.98 ± 1.81	7.72 ± 0.89	0.007
health education	7.17 ± 1.52	7.28 ± 1.15	0.261
communication skills	6.21 ± 0.77	7.44 ± 1.03	0.000
overall clinical competency	6.03 ± 1.14	7.55 ± 1.21	0.008

Note: Independent sample t test is used in Table 4

## Discussion

The standardized resident training program in China serves as a crucial component for the ongoing education and system-based clinical work training of students who have successfully finished fundamental theoretical coursework at a medical college [6]. Obstetrics is distinguished by its dynamic clinical developments and encompassing complexities, necessitating a significant level of pragmatism and feasibility [7]. Consequently, it is of paramount importance to provide obstetrics residents with comprehensive training in clinical thinking ability and skill operation ability. In China, the traditional approach to educate residents encompasses several components, including the utilization of textbooks, participation in grand rounds, and the acquisition of knowledge through the guidance of experienced specialists [8]. This education pattern tends to produce only passive learning and limited participation. Furthermore, the traditional pedagogical curriculum places emphasis on developing individual fundamental operational skills, while neglecting to provide complete instruction for the trainee’s adaptive clinical proficiency [9, 10]. Accordingly, it is imperative to investigate a novel instructional framework for obstetric residency training that can enhance the effectiveness and adaptability of teaching methods, ultimately leading to an enhancement in the quality of standardized training resident in obstetric.

ALSO course was first developed by the American Academy of Family Physicians in 1991, and introduced into China in 2002. The course places emphasis on integrating theoretical and practical instruction, distinguishing itself from conventional teaching methods. It prioritizes standardized simulation of practical training and pays close attention to students’ learning outcomes. By engaging in hands-on model teaching activities, students are equipped with the necessary skills to address obstetric issues. The course aims to assist clinical practitioners in effectively managing critical, serious, and emergency obstetric cases by employing appropriate and rational approaches. The health system and medical culture of China are very different from those in other countries [9]. Although ALSO course has been validated and used in many countries for many years, this is the first study to assess its use in an obstetric residency program in China.

In this study, compared with the traditional group, the ALSO group demonstrated superior theoretical scores and exhibited greater proficiency across most assessment items in DOPS and Mini-CEX. In terms of training content, the course covered diagnosis and treatment of vaginal bleeding during third trimester, analysis of electronic fetal heart monitoring, emergency handling, in prolapse of cord, management of shoulder dystocia, etc. The entire training process presents a multi-system crossover, integrating numerous knowledge points and skills into actual clinical scenarios. The content is generally consistent with a 2019 study in Novi Sad, Serbia [11]. While there are no additional fees for our ALSO course, which makes the program more acceptable for residents with low income in China. At this stage, it is appropriate to provide obstetrics residents, who have acquired comprehensive theoretical knowledge during their undergraduate education, with training in the ALSO program. These residents may face unexpected changes in patients’ conditions upon entering the training base, and their limited personal experience may hinder their ability to manage clinical situations promptly and effectively. From the actual implementation of the training, trainees interacted positively with the instructor and among trainees, boldly raised confusions, repeatedly operated and practiced, and showed enthusiastic enthusiasm and interest in learning. Based on the training impact observed in both theoretical and practical contexts, the trainees have acquired substantial benefits, particularly in terms of clinical reasoning and collaborative aptitude. The trainees widely acknowledge a notable enhancement in the cultivation of these two proficiencies, which can be regarded as a deficiency in conventional pedagogy and a pressing area for skill improvement among the trainees. A study in Australia showed that participants could gain a significantly increased amount of confidence and perceived knowledge as a result of completing the ALSO courses, which is similar with our findings [12]. ALSO teaching method differs from the traditional teaching method of spoon-feeding by integrating theory and practice. It emphasizes standardized and systematic practical training during the teaching process, thereby fostering the trainees’ practical problem-solving skills. This approach effectively compensates for the limitations of classroom teaching. This training model of ALSO in our study is basically in agreement with that in many developing countries and developed countries [5, 11].

The limitations of our study are that it includes a low case number and is a single-center study. A further large-scale study should be planned to confirm its application. Furthermore, this instructional approach necessitates a substantial commitment of time and effort from the clinical teachers in terms of pre-class preparation, hence creating a potential clash with the demanding clinical responsibilities. Consequently, it is imperative to increase the participation of educators in collaborative teaching efforts aimed at fostering ALSO program promotion.

## Conclusion

In summary, the utilization of ALSO course in the standardized training of obstetrics residents has the potential to significantly improve residents’ theoretical and practical performances, and ultimately enhance the overall quality of standardized training resident in obstetric.

### Electronic supplementary material

Below is the link to the electronic supplementary material.

Supplementary Material 1

## Data Availability

Data that supports the findings of this study are within the article. Further data is available from the corresponding author upon reasonable request.
