# Supplementary material for: Application of ALSO course in standardized training Resident in Obstetric
Source: BMC Med Educ. 2024 Feb 16;24:151. doi: 10.1186/s12909-024-05126-6 (PMC10874071; doi:10.1186/s12909-024-05126-6)
Supplement: Supplementary file 1 — Supplementary Material 1 [file 12909_2024_5126_MOESM1_ESM.docx]

**Application of ALSO Course in Standardized Training Resident in Obstetric**

Li Zhiyue^1^, Lu Dan^1^

^1^Clinical Medical College of Yangzhou University, Yangzhou 225001, China

Correspondence to: Lu Dan, Email: ludan1968@126.com

Table S1 Specific content of ALSO courses for the residents in the observation group

| Section number | Course content |
| --- | --- |
| Lesson 1 | Obstetric Examination and Pelvimetry |
| Lesson 2 | Vaginal Bleeding During Late Pregnancy |
| Lesson 3 | Premature Birth and Premature Rupture of Membranes |
| Lesson 4 | Analysis of Electronic Fetal Heart Rate Monitoring |
| Lesson 5 | Emergency Handling with Prolapse of Cord |
| Lesson 6 | Urgent Treatment of Eclampsia |
| Lesson 7 | Assisted Vaginal Delivery |
| Lesson 8 | Emergency Management of Shoulder Dystocia |
| Lesson 9 | Disposal Workflow of Postpartum Hemorrhage |
| Lesson 10 | Resuscitation for Pregnant and Postpartum Women |
| Lesson 11 | Asphyxia Neonatorum Resuscitation |
| Lesson 12 | Cesarean Section |

Table S2 Group comparison of DOPS score between traditional and ALSO group

|  |  | Traditional group | ALSO group | *P* value |
| --- | --- | --- | --- | --- |
| understanding of indications | to be strengthened | 2 | 1 | 0.384 |
|  | up to standard | 19 | 22 |  |
|  | excellent | 9 | 7 |  |
| obtaining informed consent | to be strengthened | 1 | 0 | 0.092 |
|  | up to standard | 23 | 20 |  |
|  | excellent | 6 | 10 |  |
| preparation of pre-procedure | to be strengthened | 19 | 1 | 0.008 |
|  | up to standard | 9 | 20 |  |
|  | excellent | 2 | 9 |  |
| appropriate level of pain relief | to be strengthened | 18 | 2 | 0.011 |
|  | up to standard | 12 | 24 |  |
|  | excellent | 0 | 4 |  |
| technical ability | to be strengthened | 17 | 2 | 0.007 |
|  | up to standard | 13 | 26 |  |
|  | excellent | 0 | 2 |  |
| aseptic technique | to be strengthened | 24 | 1 | 0.002 |
|  | up to standard | 6 | 19 |  |
|  | excellent | 0 | 10 |  |
| seeking help where appropriate | to be strengthened | 19 | 0 | 0.024 |
|  | up to standard | 10 | 24 |  |
|  | excellent | 1 | 6 |  |
| post-procedure management | to be strengthened | 14 | 1 | 0.013 |
|  | up to standard | 16 | 19 |  |
|  | excellent | 0 | 10 |  |
| communication skills | to be strengthened | 13 | 2 | 0.026 |
|  | up to standard | 15 | 20 |  |
|  | excellent | 2 | 8 |  |
| consideration of patient | to be strengthened | 19 | 3 | 0.000 |
|  | up to standard | 11 | 17 |  |
|  | excellent | 0 | 10 |  |
| overall ability to perform the procedure | to be strengthened | 15 | 0 | 0.005 |
|  | up to standard | 13 | 19 |  |
|  | excellent | 2 | 11 |  |

Note: Non-parametric rank sum test is used in Table S2.

Table S3 Group comparison of Mini-CEX score between traditional and ALSO group

|  |  | Traditional group | ALSO group | *P* value |
| --- | --- | --- | --- | --- |
| history taking | to be strengthened | 4 | 2 | 0.286 |
|  | up to standard | 21 | 16 |  |
|  | excellent | 9 | 12 |  |
| physical examination skills | to be strengthened | 7 | 2 | 0.227 |
|  | up to standard | 18 | 20 |  |
|  | excellent | 5 | 8 |  |
| organization efficiency | to be strengthened | 21 | 2 | 0.002 |
|  | up to standard | 9 | 13 |  |
|  | excellent | 0 | 15 |  |
| humanistic qualities | to be strengthened | 18 | 3 | 0.013 |
|  | up to standard | 10 | 7 |  |
|  | excellent | 2 | 20 |  |
| clinical operating ability | to be strengthened | 12 | 1 | 0.006 |
|  | up to standard | 14 | 11 |  |
|  | excellent | 4 | 18 |  |
| clinical judgement | to be strengthened | 11 | 0 | 0.020 |
|  | up to standard | 14 | 17 |  |
|  | excellent | 5 | 13 |  |
| health education | to be strengthened | 2 | 1 | 0.087 |
|  | up to standard | 12 | 11 |  |
|  | excellent | 16 | 18 |  |
| communication skills | to be strengthened | 9 | 1 | 0.026 |
|  | up to standard | 15 | 17 |  |
|  | excellent | 6 | 12 |  |
| overall clinical competency | to be strengthened | 12 | 3 | 0.114 |
|  | up to standard | 14 | 8 |  |
|  | excellent | 4 | 19 |  |

Note: Non-parametric rank sum test is used in Table S3.
